# Endogenous Fluorescence Carbon Dots Derived from Food Items

**DOI:** 10.1016/j.xinn.2020.04.009

**Published:** 2020-04-22

**Authors:** Haitao Wang, Wentao Su, Mingqian Tan

**Affiliations:** 1 School of Food Science and Technology, National Engineering Research Center of Seafood, Dalian Polytechnic University, Qinggongyuan 1, Ganjingzi District, Dalian, Liaoning 116034, China; 2 Collaborative Innovation Center of Seafood Deep Processing, Dalian Polytechnic University, Qinggongyuan 1, Ganjingzi District, Dalian, Liaoning 116034, China

**Keywords:** food-borne nanoparticles, fluorescent carbon dots, potential risk, toxicity

## Abstract

**Background:**

Fluorescent carbon dots (CDs) are a novel class of carbon-based nanomaterials that were discovered in 2004. However, nobody knew that CDs existed in food items naturally until 2012. Properties of nanosize materials are distinct from those of their bulk materials due to the particle size and accordingly alter their bioavailability and/or biocompatibility. Therefore, the potential health risk of nanoparticles in food has drawn massive attention. Currently, almost all studies regarding the biosafety of nanoparticles in food have mainly focused on engineered nanoparticles used as food additives and have excluded the endogenous nanoparticles in food. Therefore, investigation of the properties of food-borne fluorescent CDs and their potential health risk to humans is of great significance.

**Scope and approach:**

This review summarizes the existing literature on fluorescent carbon dots (CDs) in food, with particular attention to their properties, formation process, and the potential health risks posed to consumers. The knowledge gap between food-borne nanoparticles and their potential risks is identified, and future research is proposed.

**Key findings and conclusions:**

The presence of fluorescent CDs in food produced during food processing has been summarized. Fluorescent CDs less than 10 nm in size mainly contain carbon, oxygen, hydrogen, and/or nitrogen. The presence of CDs in food items was first demonstrated in 2012, and their formation was attributed to heating of the starting material. The properties of CDs in food are different from the engineered nanoparticles used as food as additives and represent a novel kind of nanostructure in food. Further studies should focus on the chronic effects of CDs, although their toxicity is low, because investigations both *in vivo* and *in vitro* are limited.

## Main Text

### Introduction

Nanomaterials ranging from 1 to 100 nm exhibit unique properties unlike bulk materials owing to their small size, quantum effects, and high surface area to volume ratio.^1^ On the one hand, these properties offer distinct benefits for the food industry. For instance, nanotechnology has been used for food quality improvement, shelf-life extension, cost reduction, and nutrition enhancement.^2^^,^^3^ On the other hand, the presence of nanomaterials in food items has raised considerable safety concerns in recent years because their specific properties may interfere with normal physiological function. The potential risk of widely used nanomaterials in food has been summarized in some previous reviews,^2^^,^^4^, ^5^, ^6^, ^7^, ^8^, ^9^, ^10^, ^11^, ^12^, ^13^, ^14^, ^15^, ^16^, ^17^, ^18^ which mainly focused on discussion of artificially engineered nanoparticles as food additives, such as nanosized Ag, SiO_2_, and TiO_2_, etc. However, nanomaterials in food items include more than just artificially engineered nanoparticles. The nanostructures derived during food thermal processing via complicated interactions among food ingredients have emerged as a new class of endogenous nanomaterials and exhibited broad potential bio-effects in the food industry.

**Figure 1:**
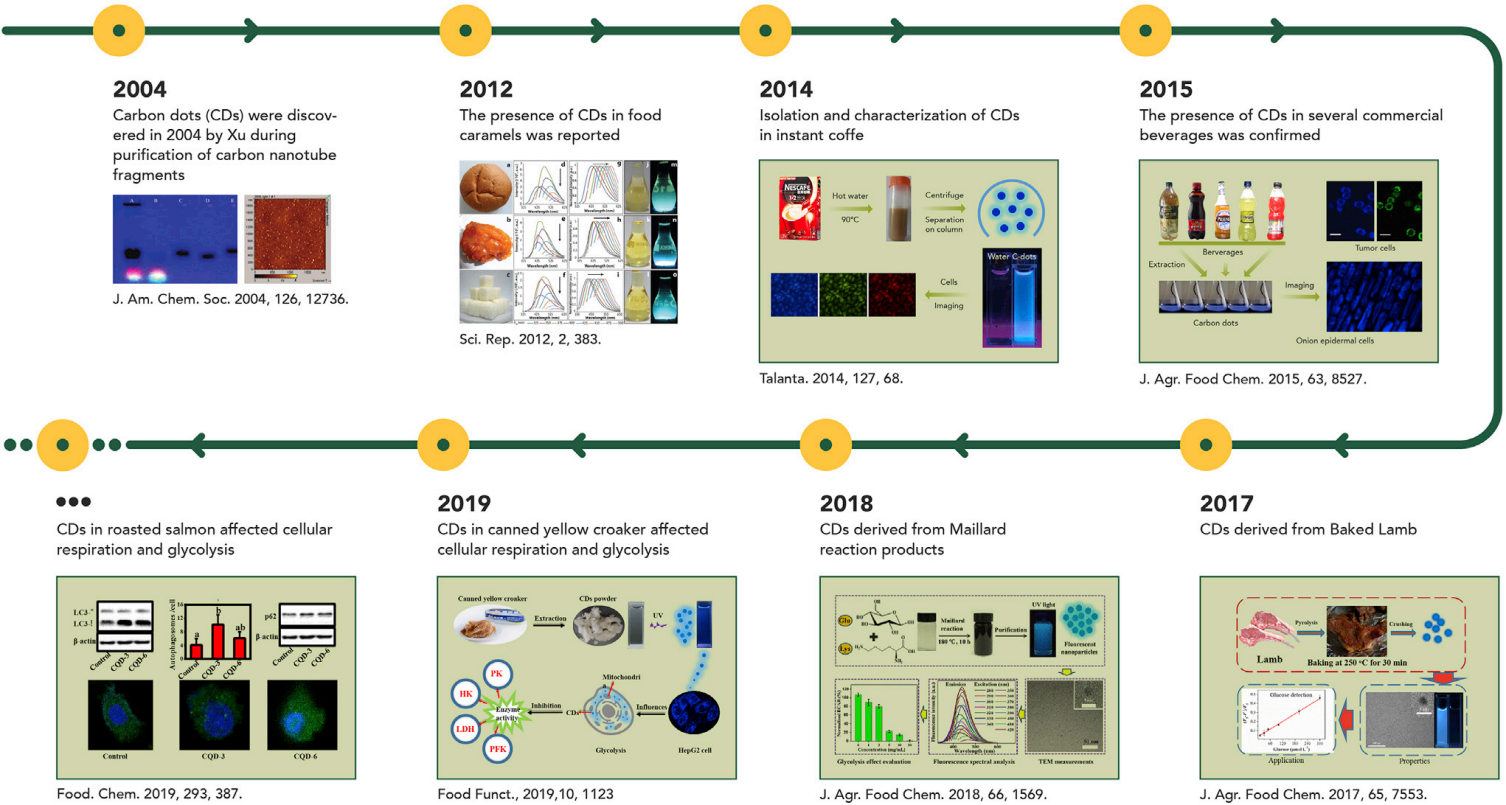
Timeline of Some Representative Events Regarding Endogenous Fluorescent Carbon Dots Derived from Food Items Reprinted with permission from Xu et al.^19^ (copyright, 2004, American Chemical Society), Sk et al.^20^ (copyright, 2012, Springer Nature), Jiang et al.^21^ (copyright, 2014, Elsevier), Liao et al. ^22^ (copyright, 2015, American Chemical Society), Wang et al.^24^ (copyright, 2017, American Chemical Society), Song et al.^25^ (copyright, 2019, Elsevier), Li et al.^26^ (copyright, 2019, The Royal Society of Chemistry), and Li et al.^27^ (copyright, 2018, American Chemical Society).

An accidental opportunity led to the discovery of carbon dots (CDs) in 2004 by Xu et al. during purification of carbon nanotube fragments,^19^ which are generally referred to as fluorescent carbon nanoparticles with an emission bathochromic shift behavior. Since that time, much effort has been devoted to the preparation of high-performance CDs, such as facile and low-cost synthesis strategies, high light-emitting capability, and renewable raw materials. However, nobody knew that CDs could be generated easily in the normal food cooking process. In 2012, Sk et al.^20^ reported the presence of CDs in food caramels, which was the first mention of CDs present in our daily food items. In 2014, Tan et al.^21^ discovered CDs that exhibited strong fluorescence in instant coffee, and in 2015, CDs were also found in certain types of beverages, such as kvass, Pony Malta, Pilsner beer, Vivant Storm, and Profit.^22^ In 2016, Al-Hadi et al.^23^ added evidence for this hypothesis by demonstrating that the CDs in bread induced metabolic stress in human mesenchymal stem cells through CYP1A and p53 gene expression *in vitro* (Figure 1). This observation suggested that nanostructures formed during the heating process may have unknown effect on the human body. Heating is the major method for food processing both in industry and at home,^28^ and almost nobody can avoid eating warmed foods. Hence, this means that humans may have been exposed to those unknown nanostructures for years. Our knowledge about the bio-effects of food-borne CDs is still in its infancy, and there is too much uncertainty about long-term exposure to food-borne CDs. Therefore, there is still a knowledge gap about the presence and properties of these emerging endogenous nanostructures, and more attention should be paid to the endogenous nanostructures produced in thermal processing owing to their potential threat to human health.

Unlike occasional exposure to engineered nanoparticles for a particular group of people, contact with these food-borne CDs is very frequent in our daily life. Generally, the physicochemical properties of nanostructures determine their acute or chronic toxicity. For nanostructures in food, understanding their long-term biological effect on humans is equally important as acute toxicity because people are likely to repeatedly take relatively low doses of nanomaterials over a long period. One reason for the health risk of nanoparticles on a human body could be attributed to their toxicity to cells. Thereby, expounding their toxicity profile to eukaryotic cells is the foundation for assessing their chronic toxicity to humans. Taking this situation into consideration, this review focuses on the endogenous fluorescent CDs produced in food items and summarizes their formation mechanism, characterization, optical properties, and their bio-effects at *in vitro* cellular and *in vivo* animal levels.

### Formation of Food-Borne CDs

Exploration of naturally occurring nanostructures in food is one of the hottest topics in the scientific community. There is much uncertainty on their effect on living organisms. CDs have a unique fluorescent property, and the discovery of novel CDs with strong fluorescence from natural sources have received considerable attention for their great potential application in bioimaging and therapy. This might be the initial motivation to find CDs in food items. To date, fluorescent CDs have been discovered in various kinds of foods items, such as roasted duck,^29^ mature vinegar,^30^ lamb chops,^24^^,^^31^ honey,^32^ pike eel,^33^ salmon,^25^^,^^34^ canned yellow croaker,^26^ beer,^35^ bread,^20^ Coca-Cola (Pepsi-Cola),^36^ kvass,^22^ coffee,^21^ grilled hamburger,^37^ pizza,^38^ caramels,^20^ and barbecue^39^. The varieties and properties of CDs from food items are summarized in Table 1. In most cases, food-borne CDs were present either in liquid food items or solid food after thermal processing at normal cooking temperatures, with a size less than 10 nm and plenty of functional groups on their surface. The emergence of these food-borne CDs in food items reminds us that natural nanostructures exist widely in our daily food, and there is not enough research to validate their biological behavior after consuming food containing CDs. The universal presence of CDs in food items has raised questions about the formation process and encourage researchers to investigate the mechanism.

**Table 1:** The Size and Surface Groups of CDs Present in Food Items

Food	Description	Size (nm)	Surface Groups	Quantum Yield (%)	Toxicity	Reference
Chicken	Heating at 200°C, 250°C, and 300°C in an oven	2.1–17.1	C=C; CO–NH	6.71–17.46	Cell viability decreased to 15%–33% after treatment with 4 mg mL^−1^ CDs	Song et al.^40^
Beer	Isolated from Snow, Harbin, Wernesgruner Dark, FAXE, and Yanjin	0.94–4.13	C=C; COOH;–OH;–NH_2_	1.42–3.92	No acute toxicity was observed in BALB/c mice after administration of a single dose of 2 g kg^−1^ body weight	Wang et al.^41^
Beer	Isolated from Tsingtao beer	1–5	–OH; –COOH; C–N–C; N–H	7.39	No obvious cytotoxicity was observed after treatment with 12.5 mg mL^−1^ CDs	Wang et al.^35^
Coffee	Isolated from Nescafe Original instant coffee	~4.4	C=C;C-O–C; COOH;–OH	5.5	No obvious cytotoxicity was observed after treatment with 20 mg mL^−1^ CDs	Jiang et al.^21^
Beverages	Isolated from Coca-Cola and Pepsi-Cola	4.7–5.0	C–O–C; C-O; C=C; –OH	3.3 and 4.3	No acute toxicity was observed in BALB/c mice after administration of a single dose of 2 g kg^−1^ body weight	Li et al.^36^
Croaker	Isolated from canned yellow croaker	1.8–5.8	C–O–C; C=O; –OH;CO–NH	9.7	Cell viability decreased to 80% after treatment with 0.125 mg mL^−1^ CDs	Li et al.^26^
Duck	Roasted at 170°C for 60 min	~1.3	C=O; C=C; CO–NH	4.4	–	Cong et al.^29^
Mature vinegar	Isolated from Chinese mature vinegar	0.5–2.5	-OH;C=O; –COOH	5.71	–	Cao et al.^30^
Glucose and lysine	Products of the Maillard reaction	2.3–6.8	O-H; O=C–O; C–O; O=C–O	16.30	Cell viability decreased to 80% after treatment with 10 mg mL^−1^ CDs	Li et al.^27^
Lamb	Heating at 200°C, 300°C, and 350°C in an oven	1.6–2.8	C=O; –NH; –OH	6–45	No obvious cytotoxicity was observed after treatment with 2 mg mL^−1^ CDs	Wang et al.^31^
Pike Eel	Heating at 160°C, 200°C, 230°C, 260°C, 300°C in an oven	1.75–4.2	C=O; C=C; C-O; –OH	12.86–80.16	No obvious cytotoxicity was observed after treatment with 20 mg mL^−1^ CDs	Bi et al.^33^
Pizza	Isolated from pizza	~3.33	C=O; –OH; –NH_2_; COOH	2.14	Cell viability decreased to 80.37% after treatment with 5 mg mL^−1^ CDs	Cong et al.^38^
Hamburger	Heating at 220°C, 260°C, 300°C in an oven	2.5–33.6	-OH; CO–NH;–NH	23.25–15.03	Cell viability decreased to 80% after treatment with 3.2 mg mL^−1^ CDs	Li et al.^37^
Beverages	Isolated from kvass, Pony Malta, Profit, etc.	5–39	-OH; C=O; C=C; C–O–C	1.48–11.9	No obvious cytotoxicity was observed after treatment with 20 mg mL^−1^ CDs	Liao et al.^22^
Atlantic salmon	Heating at 200°C in an oven	2.4–3.7	C=O; C=C; C–N	2.21–12.09	Cell viability decreased to 34% after treatment with 6 mg mL^−1^ CDs	Song et al.^25^
Honey	Isolated from honey	~3.2	C=C; –OH; –COOH	1.6	–	Mandani et al.^32^
Bakery	Isolated from bakery products	5–20	–	–	Cell viability decreased to 80% after treatment with 0.4 mg mL^−1^ CDs	Al-Hadi et al.^23^
Caramels	Isolated from caramels	4.3–27.5	–	0.63–1.2	–	Sk et al.^20^
Beef Broth	Isolated from beef broth	2.4–5.4	-OH; C=O; C=C	2.0–2.5	–	Geng et al.^42^
Mackerel	Heating at 230°C in an oven	0.9–3.5	-OH; C=O; C-N	12	–	Li et al.^43^

Progress in the synthesis methods for engineered CDs has been highlighted in a number of excellent reviews.^44^^,^^45^ Generally speaking, the strategies for CDs synthesis include two approaches, namely top-down and bottom-up methods, and some other synthetic strategies have combined those two methods. In the top-down method, macroscopic or bulk materials serve as carbon sources for CD synthesis, whereas in the bottom-up method, small organic compounds are polymerized and eventually form the carbon core of CDs under specific conditions. Most reviews emphasize the preparation and application of CDs. However, the formation of CDs from food-related material has mostly been ignored. In some cases, analogous conditions that are needed for CD formation can be formed in food thermal processing, thus producing CDs in normal cooking.

#### Normal Cooking Methods

The common methods for food processing (baking, roasting, and grilling) are all widely used for cooking food, which might form conditions similar to pyrolytic decomposition. Direct heating leads to pyrolytic decomposition of food components, thus producing black carbon that contains fluorescent CDs at higher temperature. For instance, Ye et al.^46^ reported the formation of nitrogen and sulfur co-doped fluorescent CDs via direct pyrolysis carbonization of egg. Wang et al.^47^ described a plasma-triggered pyrolytic decomposition of egg white and yolk content CDs with different properties after being irradiated by a plasma beam. Hu et al.^48^ introduced a facile method for producing sulfur-doped CDs based on pyrolysis at 100°C by using frying oil as precursor. Moreover, the formation of CDs from bread reported by Sk et al.^20^ was also a good example attributed to the pyrolytic reaction between food additive and starch during baking. Recently, our group has proven the universal formation of CDs in roast protein foods, including beef patties,^37^ grilled fish,^33^^,^^49^ baked lamb,^24^^,^^31^ popular pizza,^38^ roasted duck,^29^ and chicken.^40^ All the results demonstrated that CDs can be easily produced in normal thermal processing of foods.

#### Hydrothermal Method

The hydrothermal method refers to the treatment of food or its components in hot aqueous solutions under high vapor pressures.^50^^,^^51^ Many food ingredients have been proven to be excellent starting materials for generating CDs.^47^ For instance, Yang et al.^52^ confirmed that glucose can be converted into CDs after hydrothermal treatment, and their optical properties can be tuned simply by changing the concentration of monopotassium phosphate. Zhou et al.^53^ reported that citric acid could be used as a raw material for producing CDs. Meanwhile, the Maillard reaction, also known as non-enzymatic browning, is a chemical reaction between nucleophilic amino groups of amino acids and the carbonyl groups of reducing sugars. By using glucose and amino acids as starting materials, Wei et al.^54^ proved that all amino acids present in food except proline, cysteine, and methionine, could form CDs with glucose through the Maillard reaction. Since Maillard reactions are a prevalent chemical reaction during food processing, these results provide systematic evidence that food ingredients can generate CDs during normal food processing.

Not only small organic food ingredients can form CDs through stepwise polymerization in the bottom-up method; macromolecules in food (e.g., protein and polysaccharide) can also serve as carbon and nitrogen sources for generating CDs through the top-down method. Wang et al.^55^ demonstrated that hydrothermal treatment of milk led to the production of monodispersed, highly fluorescent CDs about 3 nm in size. Moreover, a one-step hydrothermal treatment was proven as an effective method to produce CDs from orange juice, demonstrating that a complex mixture of food macromolecules can form CDs under thermal treatment.^56^ Another example was apple juice, which can also serve as a carbon source for CD production through the hydrothermal method.^57^ Besides juice, some solid foods have also been demonstrated to be a good carbon source for CD preparation in the hydrothermal method. Song et al.^58^ developed a fluorescent probe platform based on CDs derived from black tea. Alam et al.^59^ chose cabbage as starting material and synthesized CDs with down- and up-conversion photoluminescence properties. All the results reveal that food or its components can be converted into CDs via the hydrothermal method in a facile and convenient manner.

#### Microwave Heating Methods

Unlike hydrothermal carbonization or the pyrolytic reaction during thermal processing of foods, microwave heating is often used to provide energy input in food processing. Due to the transient heating properties, the microwave technique can improve the product properties significantly, which has been proven to be an efficient method for generating CDs.^60^ There are several reports about the generation of CDs from food after microwave treatment. Wang et al.^61^ reported the formation of fluorescent CDs from milk by microwave cooking. Monte-Filho et al.^62^ revealed the production of CDs derived from lemon and onion after microwave treatment, which added further evidence to support the conclusion that microwave heating leads to the formation of CDs from food items.

#### Formation Mechanism of CDs

**Figure 2:**
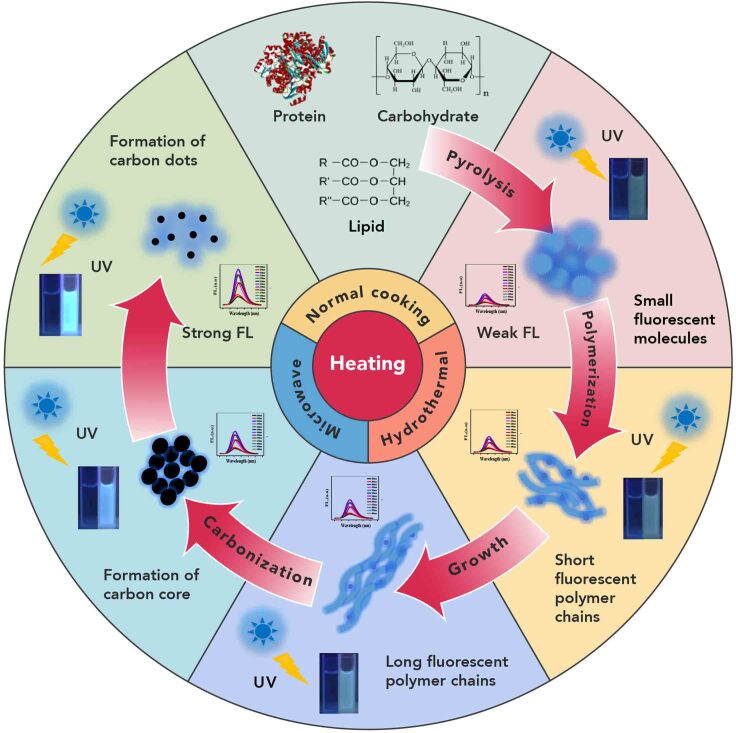
Possible Formation Mechanism of Endogenous CDs in Food Items Food ingredients undergo pyrolysis at the initial stage of heating, and small fluorescent molecules form. Short and long fluorescent polymer chains emerge in succession at the following heating stage. Fluorescent polymers further condense under heating conditions, leading to carbon core formation. The large carbon core breaks down to a small carbon core at the last stage, and carbon dots that emit strong fluorescence are formed.

The studies on the formation mechanism of CDs in food items are still at an early stage, although some preliminary results have been published.^33^ The formation of CDs is a complicated process, which involves a complex interaction among the food components and internal environments (Figure 2). Chen et al.^63^ developed a pyrolysis method to prepare CDs using natural scales and collagen powders as the carbon source. The main compounds of natural scales are collagen, a protein with a triple-helix structure. They inferred destruction of protein structures and subsequent carbonization was involved in the CD formation process. Wang et al.^47^ investigated the formation process of egg-derived CDs using thermogravimetric analysis and Fourier transform infrared spectroscopy. They proposed that proteins in the egg were denatured by high-temperature plasma in the initial stage, and then the CDs were generated through carbonization and oxidization of the precursor. Using citric acid and ammonia as an example, the possible formation mechanisms of CDs were discussed by Zhang et al.^64^ The starting materials reacted with each other and formed small fluorescent molecules through condensation at the initial stage. With the increase in temperature and extension of the reaction time, carbon cores were generated due to the increased degree of condensation and eventually formed CDs.

The facts described above suggest that food ingredients, no matter whether small molecules or large polymers, can generate CDs under normal food processing conditions through a train of complex reactions. Energy inputs, such as heating or irradiation produced during food processing, provided the initial driving force for the interactions between food ingredients and triggered the subsequent condensation and carbonization. Therefore, thermal treatment during food processing was the basis for the generation of CDs, and the parameters during food processing and the food composition are key factors that determine the properties of CDs in food items.

It is common sense that even slight changes in chemical reaction conditions may lead to significant variation of the end products. This may be equally valid for the production of CDs during food processing. Thus, the properties of CDs related to food items may be different. To fully understand the global knowledge about food-borne CDs and to maintain the focus of this article, the starting materials for CD formation discussed in this article are strictly restricted to food-related items.

### Properties of CDs in Food Items

Several factors can affect the properties of CDs, including different food components (e.g., protein, polysaccharide, lipid, vitamins, and various small molecules), synthesis method, reaction parameters, and so on. As described above, the CDs in food items can be attribute to the products of energy inputs during food processing, and the precursors for CDs are different as well. The conditions for the synthesis for typical CDs are distinct by using defined molecules as raw material. On the other hand, the formation process of CDs, no matter whether they originate from food or artificial synthesis, all involve energy input and structure conversion via a complex reaction processes, such as condensation and carbonization. Therefore, it is of great significance to summarize the properties of CDs in food.

#### Elemental Components

Food-borne CDs typically contain carbon, oxygen, and hydrogen, and carbon is usually the major constituent.^65^^,^^66^ Regarding CDs derived from protein food, they usually contain nitrogen or sulfur from food ingredients like protein. For instance, the CDs obtained from baked lamb contain 1.77% nitrogen.^22^^,^^24^ The nitrogen content of CDs derived from bovine serum albumin (BSA) was up to 14% when using BSA as a carbon and nitrogen source for CD synthesis.^67^ CDs originating from pomelo were co-doped with nitrogen and sulfur, and their content in CDs was 4.26% and 3.22%, respectively.^68^ The nitrogen content of kvass CDs was 3.0%.^22^

#### Structures and Surface Groups

The particle size of CDs from different sources varied in light of the food matrix, cooking time, and heating temperature. Some CDs from food items were extremely large; for example, the size of bread and jaggery CDs reached about 20 nm,^20^ whereas the diameter of the CDs obtained from pork was only about 3.5 nm.^69^ The size of CDs present in Tsingtao beer was between 2 and 3 nm with an average diameter of 2.5 nm.^35^

Generally, food-borne CDs were amorphous, and X-ray diffraction patterns displayed a broad peak centered at about 20°.^62^^,^^70^, ^71^, ^72^ Notably, high-resolution transmission electron microscopy observations suggested the presence of lattice spacing in CDs in some cases.^69^ These results reveal that CDs may have both amorphous and crystalline zones. The results from Raman spectroscopy suggested that CDs in a grilled hamburger consist of both sp^2^ and sp^3^ carbon sections.^37^ In addition, hydroxyl and carboxyl are common chemical groups on the surface of CDs. With heteroatom doping, amino or carbon-sulfur bonds may also exist. For instance, the CDs synthesized from casein contained sulfite and amino groups.^73^ X-ray photoelectron spectroscopy measurements indicated that the CDs from roast duck are composed of carbon (70.48%), nitrogen (6.25%), oxygen (22.17%), and sulfur (1.11%).

#### Optical Properties

Fluorescence is one of the most prominent features of CDs. Replacing toxic semiconductor quantum dots in biomedical applications was the initial motivation for investigation of CDs.^74^^,^^75^ Although yellow and red emission CDs have been reported, the emission wavelength of CDs falls mostly in the blue or green region.^76^, ^77^, ^78^ Potato CDs emit blue fluorescence, while the emission spectra of ginger CDs range from blue to green.^79^^,^^80^ The emission of CDs is affected by several factors. Theoretical modeling and experimental results suggested that size, surface groups, heteroatom doping, and defects all played roles in the fluorescence properties.^67^^,^^81^^,^^82^ Generally, the CDs showed a typical excitation-dependent emission behavior, called the bathochromic emission phenomenon. The CDs derived from food have the same characteristics.^22^ Liao et al.^22^ extracted CDs from beverages showing a fluorescence range of 430–450 nm when excited by light around 300–480 nm. CDs obtained from bread had maximum emission intensity when the excitation wavelength was 375 nm, and further increment in the excitation wavelength resulted in a red- shift of the emission maxima.^20^ The mechanisms of tunable fluorescence properties of CDs are not fully understood yet. A possible explanation is the non-uniform nature of CDs because the crystalline properties and size are different as observed by electron microscopy.^36^^,^^76^^,^^83^

Quantum yield (QY) determines the ratio of the number of photons emitted to those absorbed by the CDs. In most cases, the QY of CDs from food items is relatively low. For instance, Mandani et al.^32^ reported that the QY in honey CDs was 1.6%, and the QYs of CDs extracted from bread, jaggery, and sugar caramel were lower than 1.2%.^20^ In addition, the QY of CDs obtained from beverages was also low; for example, the QY was 5.5% for of beer CDs and 2.2% for kvass.^21^^,^^22^ The QY of CDs was related to the starting materials and synthesis conditions. However, food composition and processing conditions can significantly affect the QY of CDs in food items.^84^ Wei et al.^54^ reported that the CDs obtained from different amino acids show distinct QYs during a non-enzymatic-browning-reaction. Furthermore, CDs in real food systems provided further evidence. Biet al.^33^ mentioned that the QY of CDs from roasted pike eel increased with the processing temperature, and the maximum QY was 80.16%. Unlike CDs from roasted pike eel, the QY of CDs in grilled hamburger was much lower under the same processing temperature. This may be related to ingredient-induced differences in surface trapping excitons.^33^^,^^37^^,^^84^

Typically, the fluorescence lifetime of most of CDs is in the nanosecond range. For example, the lifetime of CDs from microwave cooked milk was about 5 ns.^61^ Similarly, Coke and Pepsi CDs had a lifetime of 4.32 and 4.26 ns.^36^ Recently, CDs with ultralong-lifetime under ambient temperature have been developed using ethanolamine and phosphoric acid as precursors.^85^ Importantly, food ingredients can also serve as starting materials for phosphorescent CD synthesis. Gao et al.^86^ obtained room temperature phosphorescent CDs successfully using aspartic acid and glucose as the starting materials. The lifetime was up to 747 ms under 320 nm excitation. The polymerized chains attached to the surface of CDs, which blocked air and moisture interference, may play an essential role in the production of phosphorescence.

#### Biodistribution of Food-Borne CDs

Biodistribution of food-borne CDs is an important issue regarding their safety concerns and potential bio-effects. Intimate knowledge about the transport, accumulation, and clearance of nanoparticles is critical to understand their behavior and outcome in biological systems.^87^, ^88^, ^89^, ^90^ The prominent features of CDs are their fluorescence, ultra-small size, and high permeability, thus providing uncertainties about their fate in the human body. Consequently, numerous reports regarding the biodistribution of CDs as potential fluorescent imaging agents have been reported. Unlike artificially synthesized CDs, the initial driving force to investigate the distribution of CDs from food items was to reveal their safety and potential toxicity risk. However, insufficient information is available on food-borne CD biodistribution, and more attention is focused on the fate of food-borne CDs after oral exposure.

##### *In Vitro* Distribution

**Figure 3:**
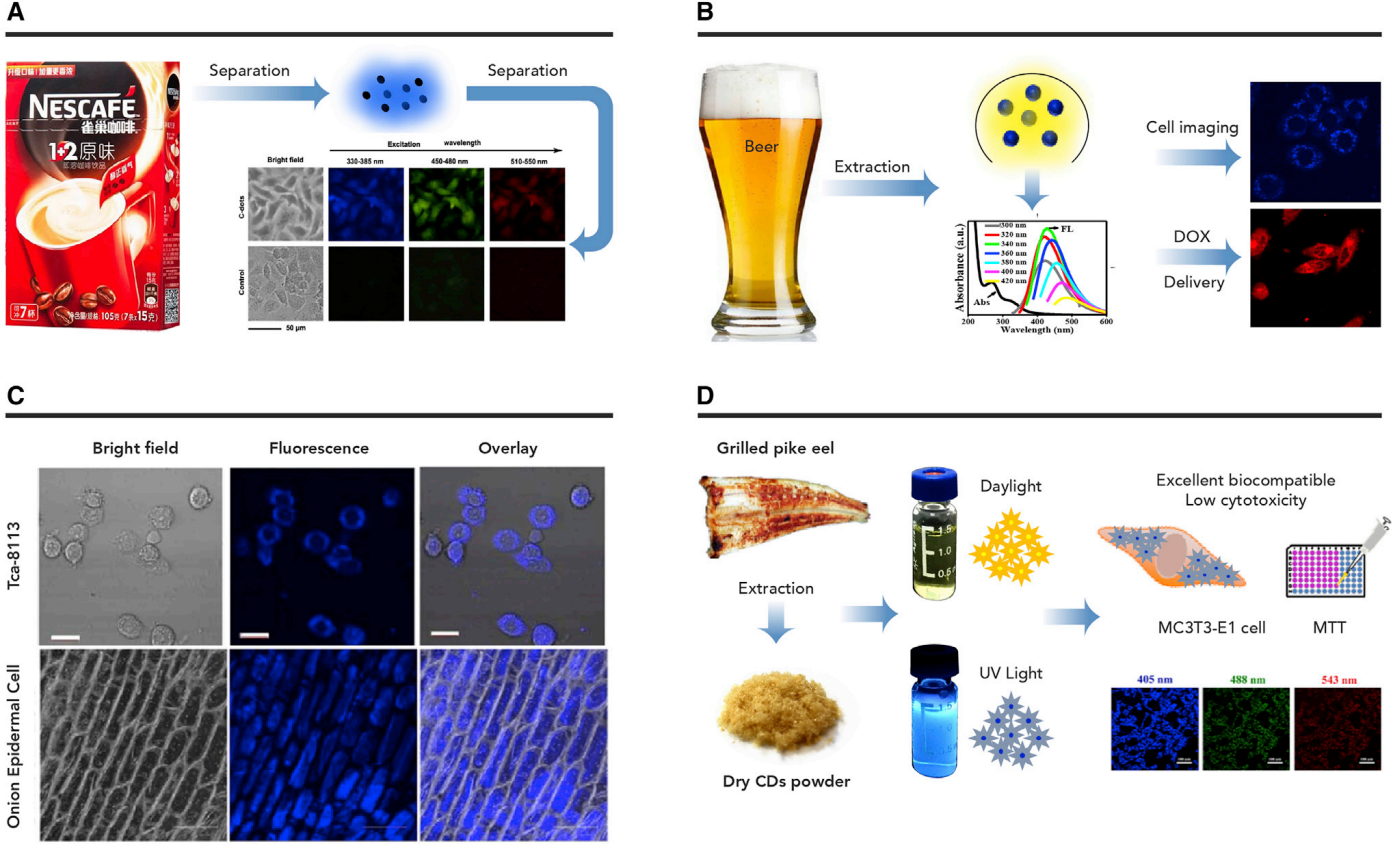
*In Vitro* Distribution of Endogenous CDs Derived from Food Items (A) Distribution of CDs from Nescafe in human hepatocellular carcinoma cells. Reprinted with permission from Jiang et al.^21^ (copyright, 2014, Elsevier). (B) Distribution of CDs from Tsingtao beer in breast cancer cells.^35^ Reproduced by permission of The Royal Society of Chemistry (Copyright, 2015, The Royal Society of Chemistry). (C) Distribution of CDs from kvass in Tca-8113 and onion epidermal cells. Reprinted with permission from Liao et al.^22^ (copyright, 2015, American Chemical Society). (D) Distribution of CDs from grilled fish in MC3T3-E1 cells.^49^ Reproduced by permission of The Royal Society of Chemistry (RSC) on behalf of the Centre National de la Recherche Scientifique (CNRS) and the RSC (copyright, 2017, The Royal Society of Chemistry).

The initial information about *in vitro* biodistribution of food-borne CDs was obtained when using food-borne CDs as fluorescent probes (Figure 3). Jiang et al.^21^ investigated *in vitro* biodistribution of coffee CDs in an SMMC-7721 cell line, and the results revealed that those cells could uptake CDs. Wang et al.^35^ explored the biodistribution of beer-derived CDs by creating an image-guided drug delivery system, and the results suggested that the beer CDs could enter different kinds of cell lines. Liao et al.^22^ extracted CDs from some commercial beverages and investigated their *in vitro* distribution. The fluorescence intensity in the cytoplasm of CD-treated Tca-8113 cells was much higher than that of the control cells, which indicated that the CDs from beverages tend to accumulate in the cytoplasm. Similar to animal cells, those CDs could also enter plant cells (onion epidermal cells) because the fluorescence was enhanced after CD treatment. In addition to CDs from liquid food, the *in vitro* biodistribution of CDs from other kinds of foods has also been investigated. For instance, the CDs from grilled fish stained both the cell membrane and the cytoplasm, but the low fluorescence intensity in the nuclear region indicated that those CDs could hardly enter the nucleus.^33^^,^^49^ The CDs formed in baked lamb were internalized by HepG2 cancer cells,^31^ whereas salmon CDs were mostly distributed within the cell cytoplasm, but the CDs were observed in the nucleus at a higher concentration.^25^

##### *In Vivo* Distribution

The behavior of CDs in food items during digestion and their metabolic fate after absorption is important to understand the impacts of food-borne CDs on biological functions. Most of the research on artificially synthesized CDs has focused on their potential applications in biomedicine, and intravenous exposure was applied during investigation of *in vivo* biodistribution of CDs.^91^ However, the biodistribution of food-borne CDs after oral exposure, especially their accumulation in major organs has not been sufficiently explored. Some *in vivo* results on animals suggested that food-borne CDs can accumulate in tissues after oral exposure. For example, Huang et al.^92^ revealed the relative low retention of CDs in the reticuloendothelial system after injection by using near-infrared fluorescence and positron emission tomography imaging. Nadia et al.^91^ carried out a sophisticated study regarding *in vivo* biodistribution of CDs in mice, which suggested a high renal clearance of CDs. Cong et al.^38^ explored the biodistribution of CDs from popular pizza in *Caenorhabditis elegans* and mice, and the pizza CDs clearly showed accumulation in the pharynx, intestine, and anus of *C. elegans* and in the gastrointestinal tract of mice. An investigation of CDs from Coca-Cola and Pepsi-Cola showed the distribution of CDs in liver, heart, brain, spleen, kidneys, intestine, stomach, and lung after oral administration in a mice model.^36^ It was notable that the CDs could cross the blood-brain barrier (BBB). In addition, the CDs derived from glutamic acid and glucose could also enter the brain after injection.^93^ This was further confirmed by Zheng et al.^94^, who reported that L-aspartic acid-derived CDs could pass the BBB and target the brain. Moreover, a comprehensive investigation of the *in vivo* tissue distribution of CDs with polyethyleneglycol (PEG) modification in mice was performed. After intravenous injection, the majority of the fluorescence signals were collected from the liver, spleen, and kidneys, which indicated that the CDs were accumulated in those organs.^95^ These findings provide additional information to understand the *in vivo* distribution of food-borne CDs.

#### Toxicity Evaluation

Since the presence of CDs was first reported in real food by Sk et al.,^20^ the safety of these CDs has been further investigated, although not thoroughly. It cannot simply be conjectured they are safe because they are originate in food. The potential health risk of CDs was identified and attracted a great deal of attention for their extensive presence in food items.

##### Cytotoxicity of Engineered CDs

Several research groups have performed toxicity assessment of artificially prepared CDs. When exposed to 0.5 mg mL^−1^ CDs derived from carbon soot by a nitric acid oxidation route, more than 90% of hepatocellular carcinoma cells survived.^96^ This concentration was much higher than the level used for bioimaging, indicating their low cytotoxicity. Results from other cell lines also support these findings. The cell viability of MC3T3 was determined through a methyl thiazolyl tetrazolium (MTT) assay; cell viability after treatment with CDs from citric acid and ethylenediamine was higher than 90%.^97^ The viability of MCF-7 cells after 24 h treatment decreased slightly when the concentration of the CDs derived from phenylenediamines was up to 50 μg mL^−1^.^98^ Similar results were obtained using human neural stem cells as a model.^99^ All these results suggest that engineered CDs exhibit a relatively low health risk to the cells.

Furthermore, efforts have been expended to investigate factors that determine toxicity profiles, and the results demonstrate that surface properties play a critical role in cytotoxicity. Havrdova et al.^100^ performed a detailed comparison among CDs with different surface functional groups using cell-cycle analysis. The results suggested that CDs modified by PEG showed the lowest toxicity because no abnormalities were observed even at concentrations up to 300 μg mL^−1^. Different from PEG-modified CDs, pristine modified CDs result in oxidative stress in a standard mouse fibroblast (NIH-3T3) cell line and arrested the cell cycle. Similarly, CDs functionalized with polyethyleneimine caused drastic changes in the G_0_/G_1_ phase of the cell cycle at a relatively low concentration.

##### Cytotoxicity of Food-Borne CDs

**Figure 4:**
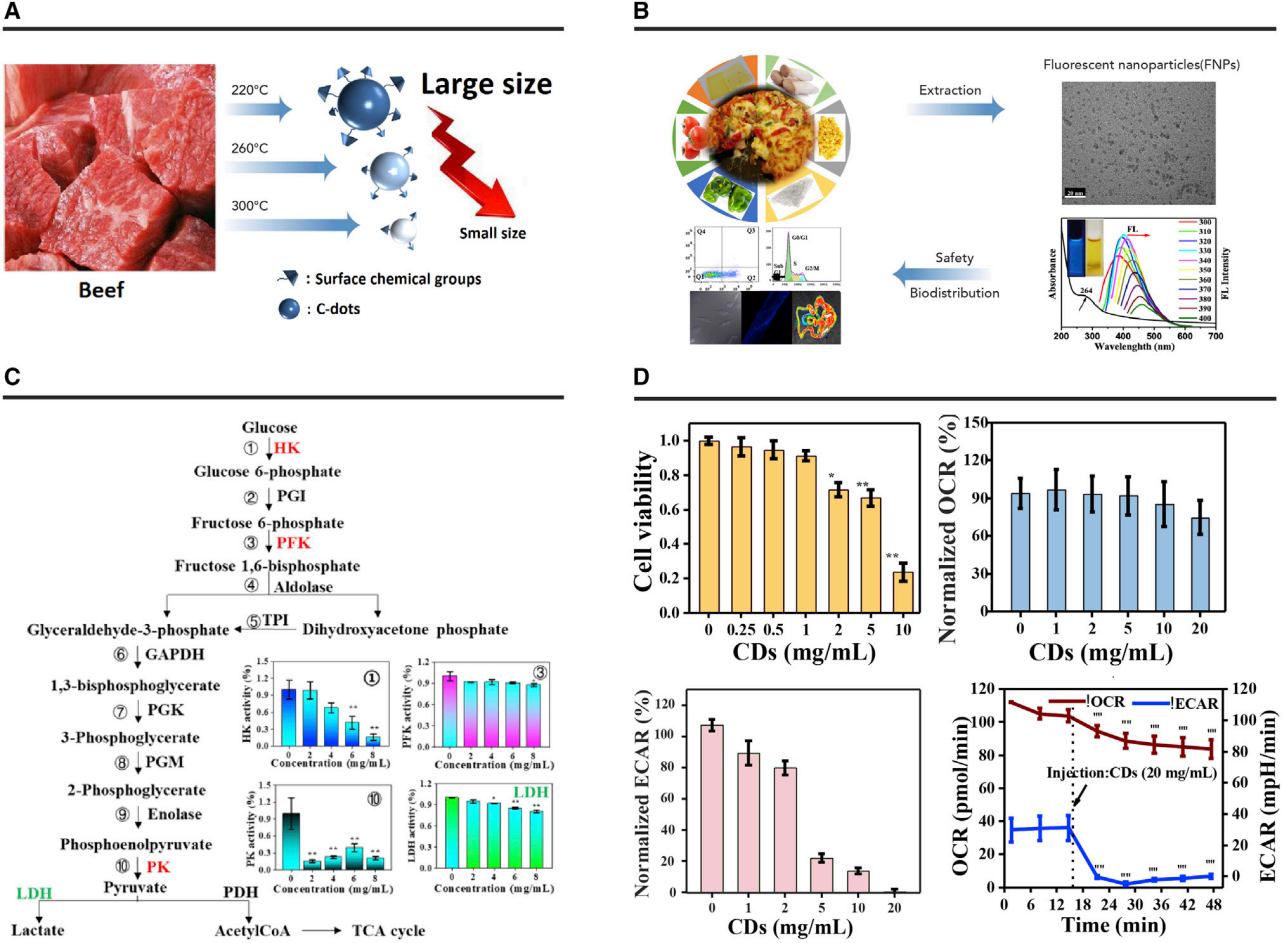
Safety Assessment of Food-Borne CDs (A) Cytotoxicity of the CDs from baked beef.^37^ Reproduced by permission of The Royal Society of Chemistry (copyright, 2017, The Royal Society of Chemistry). (B) The effects of CDs from pizza on cell apoptosis and the cell cycle.^38^ Reproduced by permission of The Royal Society of Chemistry (copyright, 2019, The Royal Society of Chemistry). (C) The effects of CDs from canned yellow croaker on enzyme activity of hexokinase (HK), phosphofructokinase (PFK), pyruvate kinase (PK), and lactate dehydrogenase.^26^ Reproduced by permission of The Royal Society of Chemistry (copyright, 2019, The Royal Society of Chemistry). (D) Cytotoxicity of the CDs from the Maillard reaction assessed by MTT assay and an extracellular flux analyzer. Reprinted with permission from Li et al.^27^ (copyright, 2018, American Chemical Society).

Considering the complexity and diversity of the precursors for food-borne CDs and the distinct formation conditions compared with engineered CDs, their surface groups are different, which in turn may lead to a distinct toxicity profile of food-borne CDs. Consequently, the safety of food-borne CDs should be assessed extensively (Figure 4). In 2014, Al-Hadi et al.^23^ performed a systematic *in vitro* assessment of CDs from bread using human mesenchymal stem cells, and the MTT assay results suggested that CDs formed during bread baking could induce metabolic stress at a high concentration. The level of intracellular reactive oxygen species (ROS) increased when exposed to bread CDs, and the mitochondrial membrane was disrupted. In addition, the transcriptional level of genes related to ROS was significantly changed. The cytotoxicity of CDs from kvass beverages was determined by MTT assay.^22^ The results suggested that cell viability was almost unaffected when the concentration of CDs was lower than 20 mg mL^−1^. The CDs from coffee showed a similar toxicity profile when using a CHO cell line as a model.^21^ The results provide primary information regarding the safety of CDs from food items.

Li et al.^37^ investigated the effects of processing conditions on the cytotoxicity of food-borne CDs from grilled hamburger at different temperatures by MTT assay. The results indicated that CDs obtained at high temperature exhibited higher toxicity. The difference in cytotoxicity might be attributed to different degrees of carbonization and surface groups of CDs at different temperatures. By using HepG2 cells as a model, the potential health risk of CDs from roasted chicken was determined.^40^ As expected, the cytotoxicity of CDs derived from roasted chicken was dose dependent, and the cell viability exhibited a decreasing trend as the roasting temperature increased from 200°C to 300°C. This result further confirms the results from grilled hamburger. Additional evidence was provided by a study on the cytotoxicity of CDs from baked lamb.^31^ The results from Annexin V-fluorescein isothiocyanate/propidium iodide double staining showed that the early apoptotic rate of cells after CD treatment was dependent on the baking temperature; the CDs obtained at a higher baking temperature exhibited higher toxicity.

Cong et al.^38^ evaluated the cytotoxicity of CDs from pizza by analysis of the cell cycle and apoptosis. The percentage of living cells decreased from 94.3% to 87.5% and 80.3% after incubation with 1 mg mL^−1^ and 5 mg mL^−1^ pizza CDs, respectively. This indicates that pizza CDs induced cell death in a dose-dependent manner. Furthermore, treatment with pizza CDs resulted in cell-cycle arrest at the G_0_/G_1_ phase, which suggests their potential health risk. Moreover, cytotoxicity assessment of CDs from canned yellow croaker provided further evidence that support the idea that food-borne CDs interrupt cellular energy metabolism.^26^ The CDs from canned yellow croaker exhibit much higher toxicity since the percentage of cell survival was about 80% at a concentration of 0.125 mg mL^−1^. Glycolysis and oxidative phosphorylation activities of HepG2 cells were measured after incubation with various concentrations of CDs. The oxidative phosphorylation activities decreased significantly when the concentration of CDs reached 6 mg mL^−1^, and the intensity was only about 30% of the control values at a concentration of 8 mg mL^−1^. This result suggests that mitochondrial functions were disrupted. Glycolysis activity showed a similar trend after exposure to canned yellow croaker CDs. Key enzymes involved in glycolysis were determined to further understand the mechanism underlying CD-induced cytotoxicity. The activity of hexokinase decreased dramatically after exposure to CDs. Pyruvate kinase was much more sensitive to CD exposure than other key enzymes in glycolysis. The activity of pyruvate kinase was only about 20% of the control group after treatment with 2 mg mL^−1^ CDs. In conclusion, these results reveal that canned yellow croaker CDs inhibit glycolysis by affecting pyruvate kinase and hexokinase and result in cell death eventually. In addition, Li et al.^27^ demonstrated that CDs can be formed through the Maillard reaction using lysine and glucose as a model system, and their potential health risk was further evaluated using a regular MTT assay and real-time cellular respiration analysis. The MTT assay showed that cell viability decreased obviously after treatment with CDs for 24 h with concentrations higher than 1 mg mL^−1^. The cell survival rate was negatively correlated with the concentration of CDs, which is consistent with other food-borne CDs described above. Energy metabolism is critical for the cell to maintain its normal physiological function. To further understand the mechanism behind CD-induced cell death and the roles of energy metabolism during this process, intensities of glycolysis and oxidative phosphorylation of HepG2 cells were determined using an extracellular flux analyzer. The results indicated that the oxidative phosphorylation rate was largely unchanged after treatment with CDs from the Maillard reaction even at an extremely high concentration (20 mg mL^−1^). Distinct from the rate of oxidative phosphorylation, the glycolysis intensity decreased significantly when the concentration of CDs exceeded 1 mg mL^−1^, and glycolysis was almost totally prohibited at a concentration of 20 mg mL^−1^, suggesting cell death after CD treatment was related to glycolysis failure and implied that CDs might interrupt the enzymes involved in glycolysis. This result was the first report to discuss the cytotoxicity of food-borne CDs from an energy metabolism perspective.

##### Interaction of Food-Borne CDs with Biomolecules

**Figure 5:**
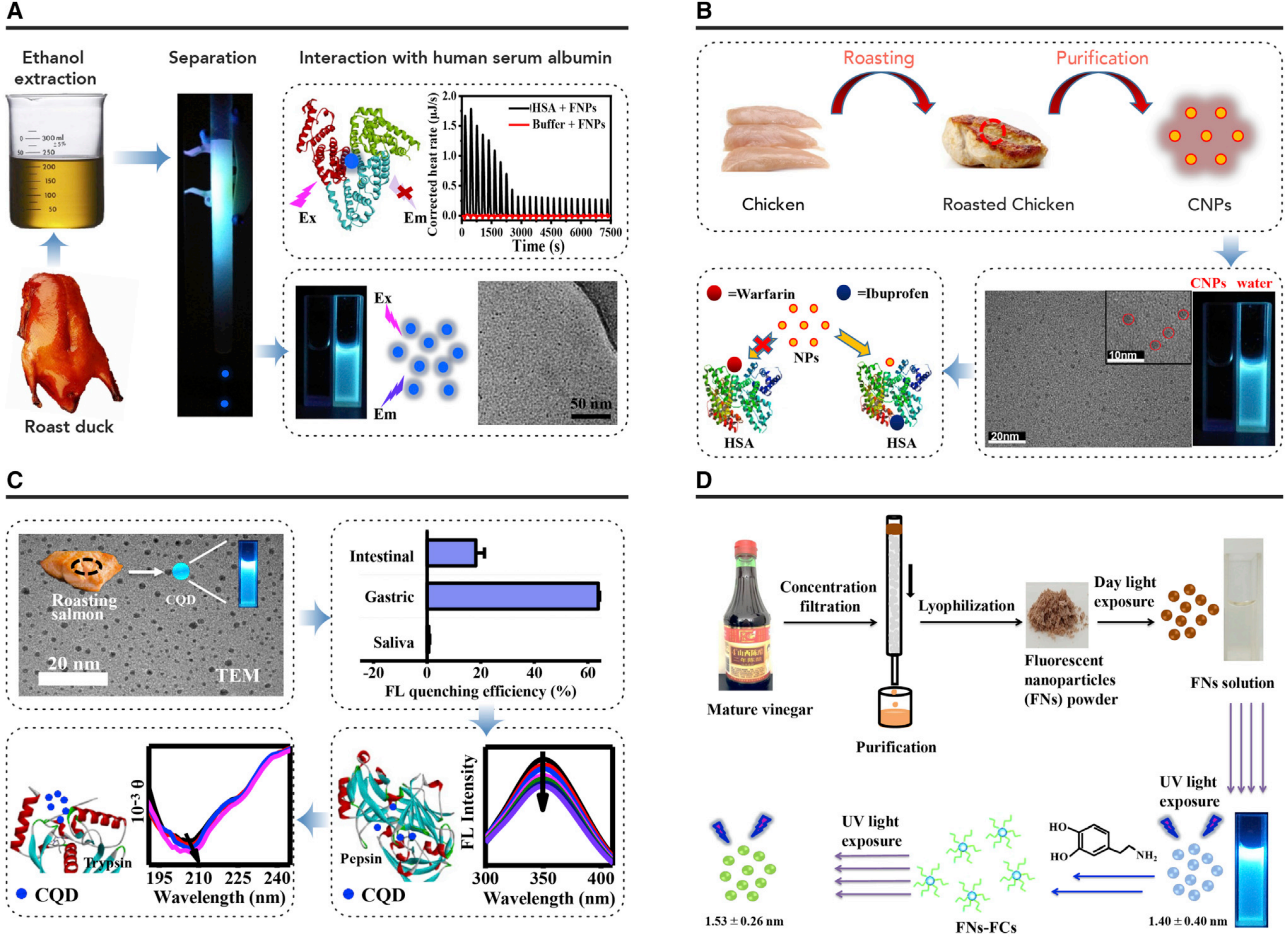
Interaction between Biomolecules and Food-Borne CDs (A) The interaction of CDs from roast duck with human serum albumin.^29^ Reproduced by permission of The Royal Society of Chemistry (copyright, 2018, The Royal Society of Chemistry). (B) The interaction of CDs from roast chicken with human serum albumin. Reprinted with permission from Song et al.^108^ (copyright, 2018, American Chemical Society). (C) The interaction of CDs from roast fish with protease and dopamine.^34^ Reproduced by permission of The Royal Society of Chemistry (copyright, 2019, The Royal Society of Chemistry). (D) The interaction of CDs from vinegar with dopamine.^30^ Reproduced by permission of The Royal Society of Chemistry (copyright, 2017, The Royal Society of Chemistry).

The interaction between nanoparticles and biomacromolecules, such as protein, nucleic acid, and lipid, could lead to changes in the structure or function of biomacromolecules, and thus interfere with the normal biological process.^101^^,^^102^ Specifically, the interaction between CDs and biomacromolecules has been demonstrated in several reports. The aggregation of human islet amyloid polypeptide was inhibited by CDs, which was attributed to the interaction between CDs and the peptide.^103^ In addition to the effect on the structure of protein, enzyme activities were also influenced by CDs. The activity of laccase was improved significantly when phosphate-modified CDs were present.^104^ In other cases, CDs inhibited the catalytic activity of lipase, and further analysis demonstrated that CDs were acting as a non-competitive inhibitor of lipase.^105^ The CDs could interact with nucleic acid through electrostatic interaction of the positively charged bond with the major groove of nucleic acid, thus inducing their conformational change.^106^ Interaction between CDs and lipid has been demonstrated as they could insert into the lipid bilayer.^107^ Similarly, interaction between biomacromolecules and food-borne CDs was also shown recently (Figure 5). Human serum albumin (HSA) is the most abundant protein in human serum and can bind various compounds, including drugs and food additives. The interaction of HSA with CDs from roast duck has been demonstrated, and the HSA structure was altered.^36^ The duck CDs were rich in hydroxyl, carboxyl, and amino groups on their surface. Fluorescence quenching and secondary structure changes in HSA may be attributed to the strong hydrogen bond between the surface of the CDs and the residue of HSA. Interaction of CDs in roasted chicken breasts and HSA has also been investigated.^108^ The results indicated that hydrogen bonding and van der Waals forces were the main forces to stabilize the CD-HSA complex. The α-helical structure decreased in CD-HSA complex compared with HSA. This result further demonstrates the effects of food-borne CDs on protein. Furthermore, food-borne CDs also affected enzyme activity. The effects of CDs from roast fish on protease have been investigated.^34^ Both the fluorescence intensity of pepsin and trypsin decreased significantly when the CDs were present. Isothermal titration calorimetry results suggested their interaction with pepsin and trypsin was spontaneous as the Gibbs free energy was negative. More importantly, the activities of pepsin and trypsin decreased remarkably, which may be associated with a conformational change of pepsin and trypsin induced by CDs, as demonstrated by circular dichroism spectroscopy. These results improve our understanding of the interaction between food-borne CDs and proteins and show the potential consequences after interacting with food-borne CDs. In addition to proteins, interaction between food-borne CDs and organic compounds has also been proven. For instance, dopamine changed the fluorescence behavior of CDs from Chinese mature vinegar through Förster resonance energy transfer.^30^ Similar results were also observed in CDs from roasted fish.^34^ These results suggest the potential health implications of food-borne CDs.

##### *In Vivo* Toxicity

Generally, the CDs in food enter the human body through eating and drinking. Therefore, the gastrointestinal tract is the first place that CDs interact with the body. The potential health risk of CDs occurs on cells or organs after the digestion and absorption of CDs in the gastrointestinal tract. Consequently, *in vivo* toxicity assessment of food-borne CDs is important. The acute toxicity of CDs extracted from beer, Coke, and Pepsi was examined in a mouse model at a dose as high as 2.0 g/kg body weight.^25^^,^^36^ No deaths or significant clinical signs of toxicity were observed after oral administration of the beer, Coke, and Pepsi CDs. In addition, some physiological indexes, including glutamate pyruvate transaminase, aspartate aminotransferase, alkaline phosphatase, lactate dehydrogenase, urea, and creatinine levels, showed slight but not significant variation after oral administration with CDs from Coke and Pepsi. Furthermore, histopathological analysis was performed to examine potential damage of major organs in mice after oral administration. No structure or morphology change was observed. The results from chemical and hematological analysis suggest that beer, Coke, and Pepsi CDs showed negligible acute toxicity *in vivo*. It is notable that *in vitro* toxicity assessment of CDs from beer demonstrated their cytotoxicity; a high dose (4.0 mg mL^−1^) of beer CDs altered cell-cycle progression and resulted in apoptosis. The inconsistent toxicity results between *in vivo* and *in vitro* may be due to the digestion process. As demonstrated by *in vitro* digestion, the fluorescence of CDs from beer, Coke, and Pepsi decreased significantly. The fluorescence quenching of CDs indicated a change in their structures and thus altered their toxicity. However, the exact mechanisms need to be further studied.

##### Effects on Gut Bacteria

The effects of nanomaterials in food on gut bacteria have drawn considerable attention.^3^^,^^109^, ^110^, ^111^, ^112^ The interaction between nanoparticles and bacteria in the colon may result in viability changes, and consequently, alter relative compositions of bacterial species. Since gut bacteria play a critical role in maintaining human health,^113^ nanoparticles in food could result in adverse health effects through changes in the gut microbiota. To the best of our knowledge, there are no reports on the impact of CDs on gut bacteria. In contrast, the antibacterial abilities of CDs have been demonstrated.^114^ For example, the CDs synthesized through a one-step electrochemical method using vitamin C as the carbon precursor displayed a strong antibacterial activity. This kind of CD could disrupt the DNA/RNA structures of bacteria and thus inhibit their growth.^114^ Anand et al.^115^ summarized the factors that affect the antibacterial nature of CDs and the mechanism behind it. Surface groups, charge, shape, and size all affected the antimicrobial ability of CDs. More importantly, it has been proven that CDs have a strong antimicrobial activity like silver nanoparticles, which could alter the gut microbiota.^116^ Therefore, it is reasonable to infer that CDs can alter the gut microbiota, especially when they reach the colon.

### Challenges and Prospects

Nanoparticles in food have been a hot topic since potential uncertainty on health was revealed, and considerable attention has been paid to engineered nanoparticles. However, the presence of food-borne nanoparticles is rarely reported, much less their potential toxic effects. CDs are food-borne nanoparticles, and studies on their properties have increased in recent years. The presence of CDs has been demonstrated in various kinds of food, especially the production of CDs during food thermal processing. Their potential adverse health effects have also received attention. The results provide deep understanding of food-borne nanoparticles and have led to insights into their underlying health risk. Nevertheless, the characteristics of food-borne nanoparticles, with emphasis on the relationship between their properties and toxic effects, are largely unknown. Therefore, further research is urgently needed to fully reveal the nature of food-borne CDs.

First, unlike engineered nanoparticles that are used as food additives, food-borne CDs are formed during food processing. The physicochemical properties of CDs from various foods as well as their relationship to food composition and food processing conditions have not been adequately characterized although many researchers have attempted to describe the process in details during CD formation.

Second, the *in vivo* behavior of food-borne CDs still needs to be uncovered. CDs are exposed to the gastrointestinal tract before they are absorbed, and the micro-environment in the gastrointestinal tract, such as low pH in the stomach and digestive enzymes, may alter the surface properties of CDs. Primary results obtained from *in vitro* digestion suggested that nanostructural CDs may be destroyed because their fluorescence decreased significantly.^29^^,^^34^ However, we still do not know about the possible change in toxicity when CDs are changed during digestion.

Third, various methods have been used to evaluate the potential toxicity of food-borne CDs, such as physicochemical, cell culture, and animal feeding studies. Different methods may obtain contradictory results; some results from animal feeding studies indicated that the CDs from food were safety, whereas other results suggested that they are toxic.^29^^,^^34^ Therefore, a standard evaluation method needs to be set up for evaluation of CD health risks.

Fourth, humans are exposed to the CDs in food items through food consumption, implying a frequent and long period of exposure at a low dose. Therefore, the potential chronic health risks of food-borne CDs are significant in the human diet.

Overall, the food matrix may have a significant impact on food-borne CD formation and properties, because human exposure occurs simultaneously. However, the effects of the food matrix on the characteristics, *in vivo* behavior, and toxicology profile of food-borne CDs are still unknown.

### Conclusion

Food-borne CDs from various food items are summarized, which has revealed their universal existence in food items. These food-borne CDs are spherical and less than 10 nm in size, and the element composition and surface groups are food specific. Primary results indicate that CDs in food could induce adverse health effects and accumulate in various organs after oral administration. This raises concerns about the long-term safety of the CDs that people consume daily in food. At present, understanding of the *in vivo* fate and toxicity of food-borne CDs is insufficient. It is important to develop standardized methods for systematic evaluation of food-borne CDs, which should consider the nature of food-borne CDs, food matrix effects, and digestion. Further research efforts should also be focused on the chronic effects of food-borne CDs.
